# Seasonal dynamics of *Fasciola gigantica* transmission in Prafi district, Manokwari Regency, West Papua, Indonesia

**DOI:** 10.14202/vetworld.2022.2558-2564

**Published:** 2022-11-11

**Authors:** Purwaningsih Purwaningsih, John Arnold Palulungan, Angelina Novita Tethool, Noviyanti Noviyanti, Fadjar Satrija, Sri Murtini

**Affiliations:** 1 Animal Health Study Program, Faculty of Animal Husbandry, University of Papua, West Papua, Indonesia; 2 Department of Animal Infectious Diseases and Veterinary Public Health, School of Veterinary Medicine and Biomedical Sciences, IPB University, West Java, Indonesia

**Keywords:** epidemiology, *Fasciola gigantica*, seasonal dynamic, snails, transmission, West Papua

## Abstract

**Background and Aim:**

Indonesia’s farming practices are a perfect setting for establishing an infection with *Fasciola gigantica* which can result in economically detrimental. The objectives of the current study were to describe and analyze the transmission dynamics of fasciolosis (*F. gigantica*) in Prafi District, to provide information on effective control strategies and to identify risk factors associated with fasciolosis in cattle.

**Materials and Methods:**

Fecal samples were purposively collected from the rectum of 60 beef cattle in Prafi District, Manokwari Regency, West Papua Province, Indonesia. The samples were collected once a month for 8 months from April 2019 to November 2019. Furthermore, the samples were taken from two rearing system groups: 30 stall cattle and 30 cattle in a palm oil garden. The presence of *F. gigantica* eggs in the fecal samples was examined using a modified Danish Bilharziasis Laboratory technique-sedimentation. Meanwhile, the antigenic diagnosis of Fasciola in the fecal samples was analyzed using an enzyme-linked immunosorbent assay kit to perform an indirect sandwich assay on feces. Snails were collected from an irrigation canal, rice field, and palm oil garden canal around Prafi District. These snails were examined for infection with cercariae larvae of *F. gigantica* by cercarial shedding and crushing techniques.

**Results:**

The peak occurrence of *F. gigantica* infection was identified in August (65.00%) and the lowest in June (35.00%). The highest prevalence of fasciolosis in cattle was recorded in August and November (90.00%) and the lowest was in May (40.00%). Moreover, the highest prevalence of fasciolosis in cattle exposed to the palm oil garden was recorded in April (53.33%) and the lowest prevalence of *F. gigantica* infection was recorded in June (23.33%). In total, 2046 snails were screened by the cercarial shedding and crushing method; of these, 426 (20.82%) were found to be positive for trematode cercariae. The prevalence of *F. gigantica* infection in *Lymnaea* spp. snails was highest in November (47.46%) and lowest in April (9.28%).

**Conclusion:**

The current study shows that beef cattle grown in two types of rearing systems in Prafi District were infected with *F. gigantica* during the dry and rainy season. It was revealed that *Lymnaea* spp. are common snails found in and around Prafi District, and can act as intermediate hosts with an infective stage of trematode. The transmission to cattle was highly effective, despite the short activity period, the low infection rate of snails, and the incidental anthelminthic treatment.

## Introduction

Indonesia is one of the tropical countries in Southeast Asia. As a tropical country, it relies on cattle in agricultural systems in several provinces. One example is the population of 55,500 beef cattle in West Papua [1], that is, one of the provinces in Papua, the largest island in Indonesia with excellent savanna potential. Within the province, there is a focus on some subsystems, particularly crop residues when harvested (rice straw) and herbage plants from around the crop area to complete their diet. Despite the great potential as mentioned previously [1], the resources are not maximally utilized when addressing management problems in the province. One example of such a problem is the veterinary parasitic disease of ruminants caused by digenean trematodes of the genus Fasciola, *F. hepatica*, and *Fasciola gigantica* (giant liver fluke). These trematodes are commonly referred to as liver flukes [2]. Some livestock is reared under diverse agro-climatic rearing management and conditions [3]. The farming system in Indonesia provides an ideal environment for maintaining *F. gigantica* infection. It is unsurprising that fasciolosis is a common parasitic disease affecting cattle in areas with intensive rice production. This disease is economically detrimental, resulting in increased condemnation of liver meat, greater fatality rate, decreased production of meat, milk, wool, weight decline, infertility [4], and costs of animal treatment [5].

The eradication of fasciolosis in ruminants is rarely a practical option. Therefore, approaches to eliminate the disease are required [6]. Parasite epidemiology provides a basis for designing efficient and sustainable strategic control programs for domestic animals that suit the climatic conditions of each region [7]. In epidemiological studies on fasciolosis, the main risk factors involved in the transmission dynamics have been examined extensively worldwide [8–11]. The spread of fasciolosis is closely related to environmental factors, for example, temperature, humidity, rainfall, altitude, and livestock management system [12]. Moreover, fasciolosis commonly depends on the presence and ecology of the snails that act as an intermediate host [13]. These snails belong to a large and highly diverse group of invertebrates of the phylum Mollusca, the class Gastropods, the order Stylommatophora, and the genus *Lymnaea* and are involved as an intermediate host in the parasite life cycle. Snails have free-living stages comprising several developing larval stages: Eggs, miracidia, sporocysts, rediae, cercariae, and metacercariae [14]. The biology of *Lymnaea* spp. was described by Martin and Cabrera [15]. An epidemiological study of the larval stage of *F. gigantica* in snails was conducted in India [16]; however, little is known about the population dynamics of this snail in areas with extensive irrigated rice production. There are numerous reports on the prevalence of fasciolosis in different parts of Indonesia: 4.9% in Muna Regency [17], 4.4% in Bulukumba Regency [18], 17.19% in Kupang City [19], 56.3% in Aceh [20], 50.43% in Pekanbaru City [21], and 27.62% in Sukoharjo District [22]. Several studies have reported the prevalence of bovine fasciolosis in different countries: 26% in Ethiopia [23], 74.9% and 28.2% in north and central Nigeria [24, 25], 9.77% in the Nile Delta region of Egypt [26], and 65.7% in northern Uganda [27]. The prevalence of larval trematode infection in snails was 47.32% in Sigi Regency [28], 10.5% in Cuba [29], 71.8% in the West Indies [30], and 8.60% in India [31]. It is worth noting that no collaborative study has been conducted to study the transmission patterns of the disease over a considerable period.

Hence, this study investigated the epidemiological pattern of fasciolosis in different rearing systems and agro-climatic zones to provide a basis for designing strategies to control this disease. This study was designed to generate data about risk factors affecting the prevalence of fasciolosis in West Papua.

## Materials and Methods

### Ethical approval

This research does not require animal ethics approval because fecal samples were collected from the ground without harming the animals.

### Study period and location

The study was conducted from April to November 2019. This study was conducted in Udupi Hillur village **(**0°14’ s and 130°31’ e, 99 meters above the sea level), Prafi District, Manokwari Regency, West Papua Province, Indonesia. In the village, the average annual rainfall is 237.8 mm, and the temperature varies from 24.9°C to 31.4°C with a humidity of 84.1%.

### Study design and sampling

In total, 480 fecal samples were collected from randomly selected areas of Prafi District; this was planned to allow the monthly and seasonal variations of fasciolosis in beef cattle to be determined. Fecal samples were collected directly from the rectum of each animal every month (60 samples/month: 30 fecal samples from stall cattle and 30 fecal samples from free-ranging cattle). Ten grams of fecal matter were placed in a labeled zip-lock plastic bag and transported to the laboratory in a preservative, that is, 10% formalin. Sampling was conducted once a month for 8 months (from April to November 2019).

### Coprological examination

Samples were examined for the presence of Fasciola (*F. gigantica)* eggs by Danish Bilharziasis Laboratory technique (DBL)-sedimentation [32]. DBL-sedimentation was employed to perform fluke egg count (EPG) in the positive samples.

### Meteorological/climatic data

Meteorological data, including maximum and minimum temperature (°C), relative humidity (%), and rainfall (mm), were obtained from the meteorological station at Manokwari Regency. Correlations with the occurrence of fasciolosis were identified.

### Collection and identification of snails

The population of *Lymnaea* spp. snails was monitored regularly at each location, allowing the comparison of the snail population at different sites. Based on the pilot study result, snails were collected from four sites in each village: Irrigation canal, irrigation canal of the palm oil garden, dam, and rice field irrigation. The irrigation canal site was divided into ten spots: Two rice fields, one irrigation canal of the palm oil garden, and two spots in the dam. The two spots in the dam were at sites where the cattle were permitted to graze on this part. Such an approach enables the dung from the irrigation canal to enter the irrigation canal of the rice field, palm oil garden, and then the rice field. The snails were collected every 4 weeks for 8 months from April to November 2019. All snails were sent to a laboratory in plastic jars containing a small amount of tap water. Each snail was isolated in a plastic pot filled with tap water and maintained at room temperature (25.0°C ± 2.0°C) for 24 h. Water from each container was examined using a microscope to identify the presence of cercariae. Cercariae identification was based on a key for trematode cercariae [33].

### Statistical analysis

The raw data were input into a Microsoft Excel spreadsheet and then summarized by descriptive statistics. The prevalence of fasciolosis in beef cattle, snails, and cercariae was calculated as a percentage (%) and tabulated by group area, month, and season. The Chi-squared test was employed to assess the differences in the occurrence of fasciolosis between different risk factors (age, sex, body condition score, and rearing system); values of p ≤ 0.05 were considered significant. Statistical analyses were performed using Statistical Package for the Social Sciences version 24 software (IBM Corp., NY, USA).

## Results

### Climatology report for the research area

**Figure-1:**
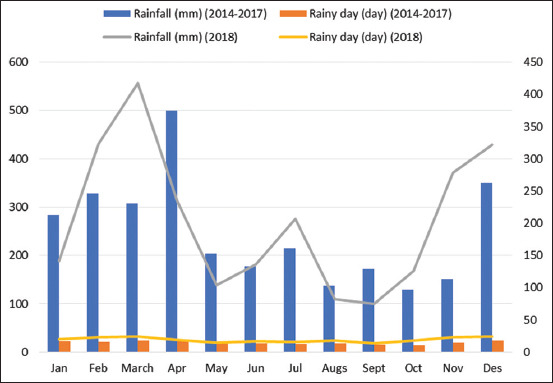
Average rainfall per month within 5 years.

The average rainfall per month for 5 years between 2014 and 2018 (Figure-1) was 237.8 mm each month. The lowest rainfall was 75.1 mm in September and the highest was 499.6 mm in April.

### Prevalence of *F. gigantica* in beef cattle

The monthly variation in the occurrence of fasciolosis in beef cattle is presented in Table-1. The overall prevalence of fasciolosis caused by liver fluke in beef cattle from April to November was 52.50%, and the mean EPG range was 0.20–1.30. The data indicate a significant difference in the occurrence of infection between different months (Table-1).

**Table-1:** Monthly prevalence of fasciolosis in Prafi district from April-November 2019.

Month	Prevalence of fasciolosis (No. of positive cases/total sample collected)			Mean egg counts (EPG)	χ^2^-value

	Stall cattle	Exposed cattle	Total		
April	16/30 (53.33)	22/30 (73.33)	38/60 (63.33)	1.10	27.19**
May	11/30 (36.67)	12/30 (40.00)	23/60 (38.33)	0.53	
June	7/30 (23.33)	14/30 (46.67)	21/60 (35.00)	0.20	
July	10/30 (33.33)	13/30 (43.33)	23/60 (38.33)	0.23	
August	12/30 (40.00)	27/30 (90.00)	39/60 (65.00)	1.30	
September	9/30 (30.00)	26/30 (86.67)	35/60 (58.33)	0.67	
October	14/30 (46.67)	21/30 (70.00)	35/60 (58.33)	1.02	
November	11/30 (36.67)	27/30 (90.00)	38 (63.33)	1.26	
Average	90/240 (37.50)	162/240 (67.50)	252/480 (52.50%)		

χ^2^ test of significance for overall month-wise comparison of *fasciolosis,*

** p<0.01, *p<0.05, NS=Non significant

The table shows a significant difference in the infection rate of *F. gigantica*. The highest prevalence was in August (65.00%) and the lowest prevalence was in June (Table-1).

### Seasonal prevalence

In the seasonal pattern of fasciolosis, the highest infection rate in exposed cattle (90.00%) was recorded in August and November 2019; the lowest (40.00%) in May. The highest prevalence in cattle was observed in April (53.33%); and the lowest was in June (Table-1). Furthermore, the monthly data show that the highest prevalence was recorded in August and the highest EPG was in August, which gradually declined in April, followed by a fall to the lowest infection in June.

### Prevalence of larval stages of *F. gigantica* in snails

**Figure-2:**
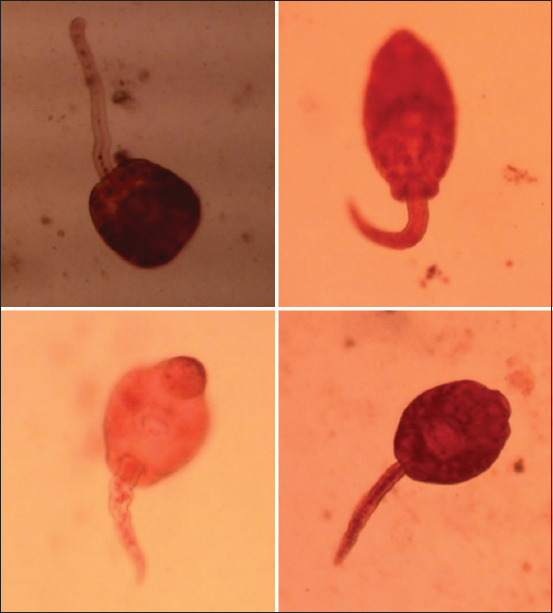
*Fasciola* spp. cercariae: Gymnocephalus type. Source: (primary data).

**Figure-3:**
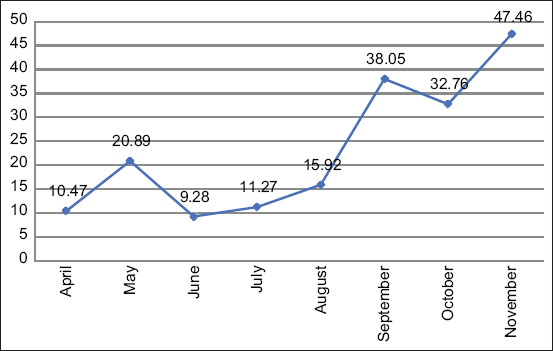
Prevalence of larval stage *Fasciola gigantica* in snails from April to November 2019.

In total, 551 *Lymnaea* spp. snails were examined to identify cercariae of *F. gigantica*. The overall prevalence of the snail’s intermediate host was infected with different cercariae trematodes. Meanwhile, 20.82% of the snails harbored cercariae of *F. gigantica* (Figure-2), with the maximum prevalence of 47.46% in November and the lowest (9.28%) in June (Figure-3).

Cercarial types were recovered on a snail belonging to a group of *Gymnocephalus cercariae*. This type of cercariae has appropriate characteristics for *Fasciola* spp. cercariae. The study of Chontananarth and Wongsawad [34] supports this study’s findings, stating that the trematode that produces this type of cercariae is from the family Fasciolidae.

*Gymnocephalus cercariae* are oval-shaped (Figure-2). They have a sub-terminal oral sucker and a short anterior pharynx. The esophagus branches postero-lateral to the ventral papilla and extends almost to the posterior quarter. The excretory vesicles are spherical. The central collecting duct rises from the anterior wall of the vesicles and extends from the sucker area of the pharynx and cheeks. The duct contains large granules. The ventral sucker is highly extensible, tends to be larger than the oral sucker, and is slightly behind the center of the body. The tail is strongly developed and about the same length as the pleated body of the dorsoventral [34].

The Chi-squared value showed that the prevalence rate of the larval stage *F. gigantica* in snails was significantly higher in September, whereas the lowest rate was in June (Table-2).

**Table-2:** Overall monthly prevalence of *Lymnaea spp.* is having infective stages of *Fasciola gigantica* in Prafi district.

Month	Number of snails collected	Number of positive snails	Prevalence (%)	χ^2^-value
April	382	40	10.47	213.514**
May	225	47	20.89	
June	194	18	9.28	
July	355	40	11.27	
August	333	53	15.92	
September	205	78	28.05	
October	116	38	32.76	
November	236	112	47.46	
Total	2.046	426	40.11	

χ^2^ test of significance for overall monthly comparison of Lymnaea spp.

** p<0.01, *p<0.05, NS=Non significant

This study further revealed that the age, sex, body condition score, and rearing system of the beef cattle had no significant correlation with the prevalence of fasciolosis (p > 0.05) (Table-3).

**Table-3:** Prevalence of fasciolosis based on the different risk factors.

Risk factors	No. of examined (%)	No. of infected (%)	p-value
Age			
Calf	85 (17.71)	56 (65.88)	0.285
Adult	395 (82.29)	196 (49.62)	
Sex			
Male	190 (39.58)	74 (38.95)	0.430
Female	290 (60.42)	178 (61.38)	
Body condition			
Poor	107 (22.29)	86 (80.37)	0.407
Medium	282 (58.75)	138 (48.94)	
Good	91 (18.96)	28 (30.77)	
Rearing system			
Stall	150 (50.00)	90 (37.50)	0.447
Exposed	150 (50.00)	162 (67.50)	
Total	480	252 (52.50)	

## Discussion

This study showed that fasciolosis is a common health problem in beef cattle in almost all areas of the Manokwari Regency, with an overall prevalence of 52.50%. This finding is higher than some reports, such as Manu Regency with 4.9% [17], Bulukumba Regency with 4.4% [18], Kupang with 17.19% [19], and Sukoharjo with 27.62% [22]. In contrast, the present prevalence was much lower than in other areas, such as 50.43% in Pekanbaru City [21] and 56.3% in Aceh [20]. In other countries, the prevalence of bovine fasciolosis varied: 74.9% in Ethiopia [23], 28.2% in north-central Nigeria [24, 25], 9.77% in Egypt [26], and 65.7% in Uganda [27]. The difference in prevalence among geographic locations is primarily connected to climatic and ecological conditions, for example, altitude, rainfall, and temperature. The prevalence of fasciolosis has been reported to vary over the years, mainly due to changes in the rainfall rate and pattern [35].

Based on the examination of the 480 feces samples from beef cattle focusing on the eggs of *F. gigantica* (Table-1), a high prevalence of fasciolosis infection was determined. The prevalence of fasciolosis fluctuates over different months, ranging from 35.00% to 65.00%. The highest prevalence of the research (65.00%) was recorded in August 2019.

The results revealed that the disease was also a problem in beef cattle at livestock smallholders in Prafi District, and caused the high economic loss. No significantly higher prevalence (p > 0.05) of fasciolosis was recorded in free-ranging cattle (67.50%) than in stall cattle (37.50%). The prevalence of fasciolosis in exposed beef cattle is higher than in stall cattle due to the equal possibility of infection when exposed to parasites in the communal grazing pasture. Such a prevalence allowed the cattle to graze and search for their food in palm oil gardens and other wastelands [36]. This is because almost all small-scale farmers practice keeping their animals for pasture grazing in groups for a long time compared with other types of farmers. Such a situation creates a suitable environment for uninfected cattle to acquire a high level of infective larvae from the infected pasture. In the extensive rearing system, beef cattle remained in the stall full-time and fed under a cut-and-carry system with forage sourced from rice banks, creek banks, and irrigation channel banks.

The prevalence of fasciolosis was 38.95% and 61.38% in male and female cattle, respectively. However, there was no statistically significant difference between the prevalence recorded in females and males (p > 0.05) (Table-2). These results are similar to several studies which reported a higher prevalence of fasciolosis in female cattle than in male cattle [37, 38]. This trend agrees with the result of studies by Aliyu *et al*. [37] and suggests that there is hormone-mediated control of immunity in female animals during pregnancy and lactation, thereby increasing their susceptibility to *F. gigantica* infection [37]. The lower prevalence of fasciolosis in males than females might be explained by the fact that adult male animals are not usually exposed to grazing in the field. Most farmers feed their male animals by tethering them around homes to fatten them [39].

In this study, the prevalence of *F. gigantica* was higher in young cattle (<2 years old) than in adults (>2 years old). This result indicated no significant difference between age group and prevalence of fasciolosis (p > 0.05) and corresponded with [35]. This may be due to the development of acquired immunity in older animals, which results in resistance, as identified in Aliyu *et al*. [37].

There was no significant difference between infection rate (p > 0.05) and body condition scores by group concerning risk factors. Further, there is a higher prevalence of fasciolosis in cattle with a poor body condition than in medium and good body conditions [40], as progressive losses characterize chronic fasciolosis.

Out of 2046 *Lymnaea* spp., 426 (20.82%) harbored the larval stages of *F. gigantica*, and the highest prevalence was found in November. A study by Oladele-Bukola and Odetokun [36] supports our findings as they reported a prevalence of cercarial infection in snails; approximately 17.27% of snails were infected by different trematode cercariae [34]. In contrast, another study reported a higher prevalence rate and found that 74.56% of snails were contaminated with other trematode cercariae in Iran [41]. A study reported a low prevalence rate of cercarial infection in snails; it is shown that 3.9% of *Lymnaea* spp. was infected by different trematode cercariae [42].

The data indicate that the percentage of infection in the cercariae stage in snails was higher between September and November (onset in rainy season) and lower in June (dry season). A study also reported that the prevalence of the infection cercariae in snails was highest during the rainy season and lowest during summer [31]. A higher infection in November may be caused by close contact between infected animals or their feces and snails. During summer, water bodies shrink, and the concentration of snails increases in small water hollows where ruminants gather for water and graze on grasses.

The occurrence of cattle fasciolosis is high in August, which may be because of close contact between infected animals or their feces and snails. Moreover, there is a shrinkage of water holes where ruminants gather for water. With the onset of the dry season in April, a new crop of snails is produced, further contributing to the high infectivity of snails in August/September. The high prevalence we observed may be due to the snail-infected parasitic stages, namely, the cercarial stage during the mid-rainy season. The egg-containing miracidium is being conducted in the water of the low-lying areas of the snail varieties.

The seasonal prevalence of fasciolosis was reported to be higher during the dry season than during the rainy season. There is a significant intensity of final host animals around drinking sites during dry seasons. Due to the absence of natural pastures, animals were forced to graze on Fasciola-encysted cercariae-contaminated plants around the canal banks.

The distribution pattern of *F. gigantica* infections from this study showed that areas with higher rainfall intensities had a higher prevalence than areas with the lowest precipitation. Such a difference in the prevalence of fasciolosis might be attributable to ecological and climatic variations in some regions and animal husbandry practices. This variation may also be due to different internal parasite control between countries.

## Conclusion

This study revealed that fasciolosis is an occurrence in beef cattle farming in the Prafi District Manokwari Regency West Papua Province, Indonesia. The common snails as an intermediate host in bovine fasciolosis are *Lymnaea* spp. The type of cercariae as an infective stadium for *F. gigantica* is *Gymnocephalus cercariae*. The host factors are age, sex, body condition score, and management practice for the beef cattle rearing system, had no significant correlation with the occurrence of fasciolosis. This study revealed that the occurrence of fasciolosis and infection of larval stages in snails were significant overall month-wise in Prafi District Manokwari Regency West Papua Province, Indonesia. Our findings provide basic epidemiological information and prevention plans for fasciolosis in beef cattle farming in West Papua Province, Indonesia.

## Authors’ Contributions

FS: Designed the study. ANT: Conducted the study. PP and JAP: Interpreted the results and drafted the manuscript: NN: Revised and finalized the manuscript. SM: Supervised the study. All authors have read and approved the final manuscript.
